# Supplementary figures and images for: The zona incerta negatively regulates the red nucleus during movement cued by sound signals
Source: PLoS Biol. 2025 Apr 7;23(4):e3003092. doi: 10.1371/journal.pbio.3003092 (PMC12002635; doi:10.1371/journal.pbio.3003092)

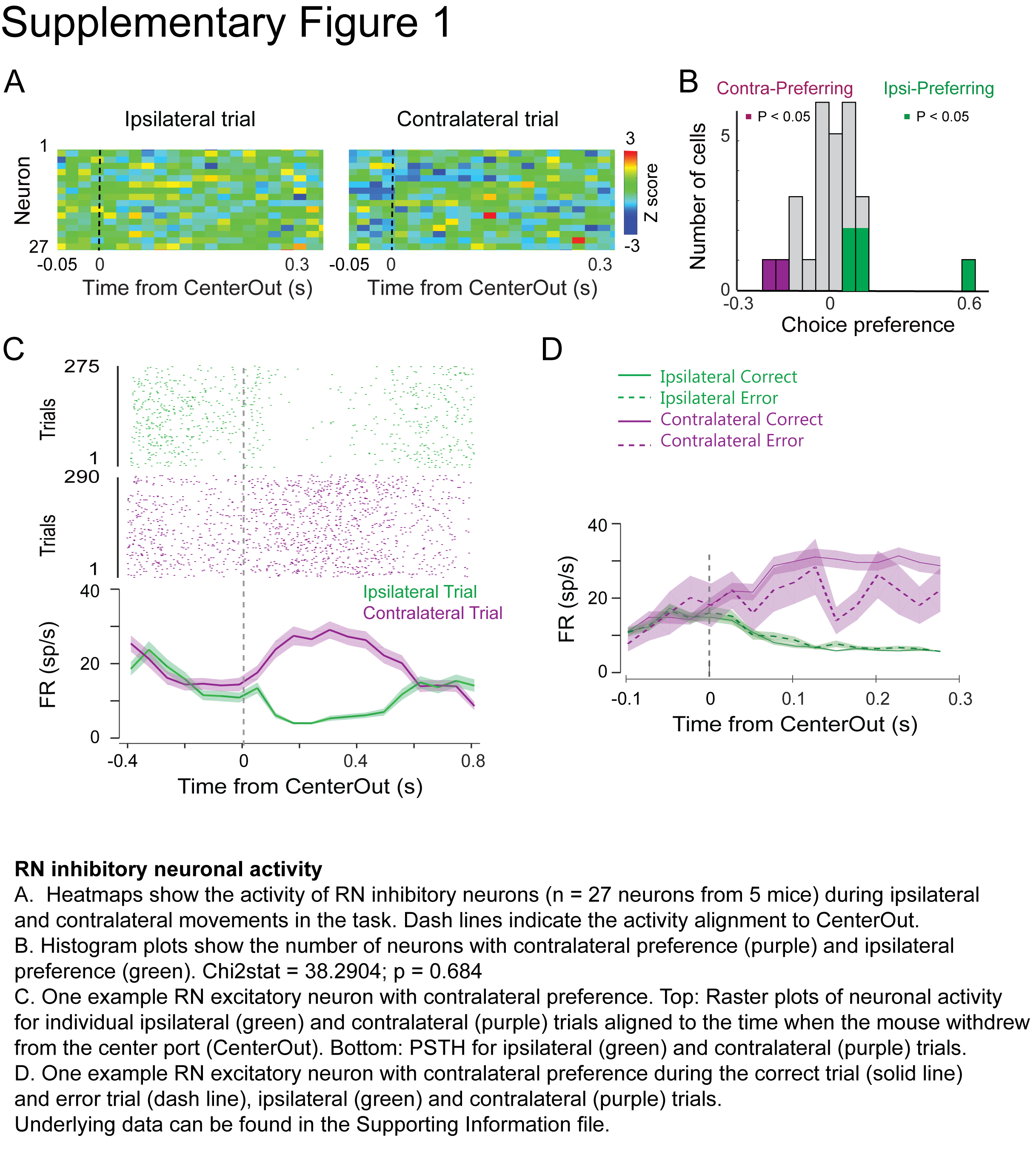

Supplement: S1 Fig — A. Heatmaps show the activity of RN inhibitory neurons (n == 27 neurons from 5 mice) during ipsilateral and contralateral movements in the task. Dash lines indicate the activity alignment to CenterOut. B. Histogram plots show the number of neurons with contralateral preference (purple) and ipsilateral preference (green). Chi2stat == 38.2904; p == 0.684. C. One example RN excitatory neuron with contralateral preference. Top: Raster plots of neuronal activity for individual ipsilateral (green) and contralateral (purple) trials aligned to the time when the mouse withdrew from the center port (CenterOut). Bottom: PSTH for ipsilateral (green) and contralateral (purple) trials. D. One example RN excitatory neuron with contralateral preference during the correct trial (solid line) and error trial (dash line), ipsilateral (green) and contralateral (purple) trials. Underlying data can be found in the S1 Data. (TIF) [file pbio.3003092.s001.tif]

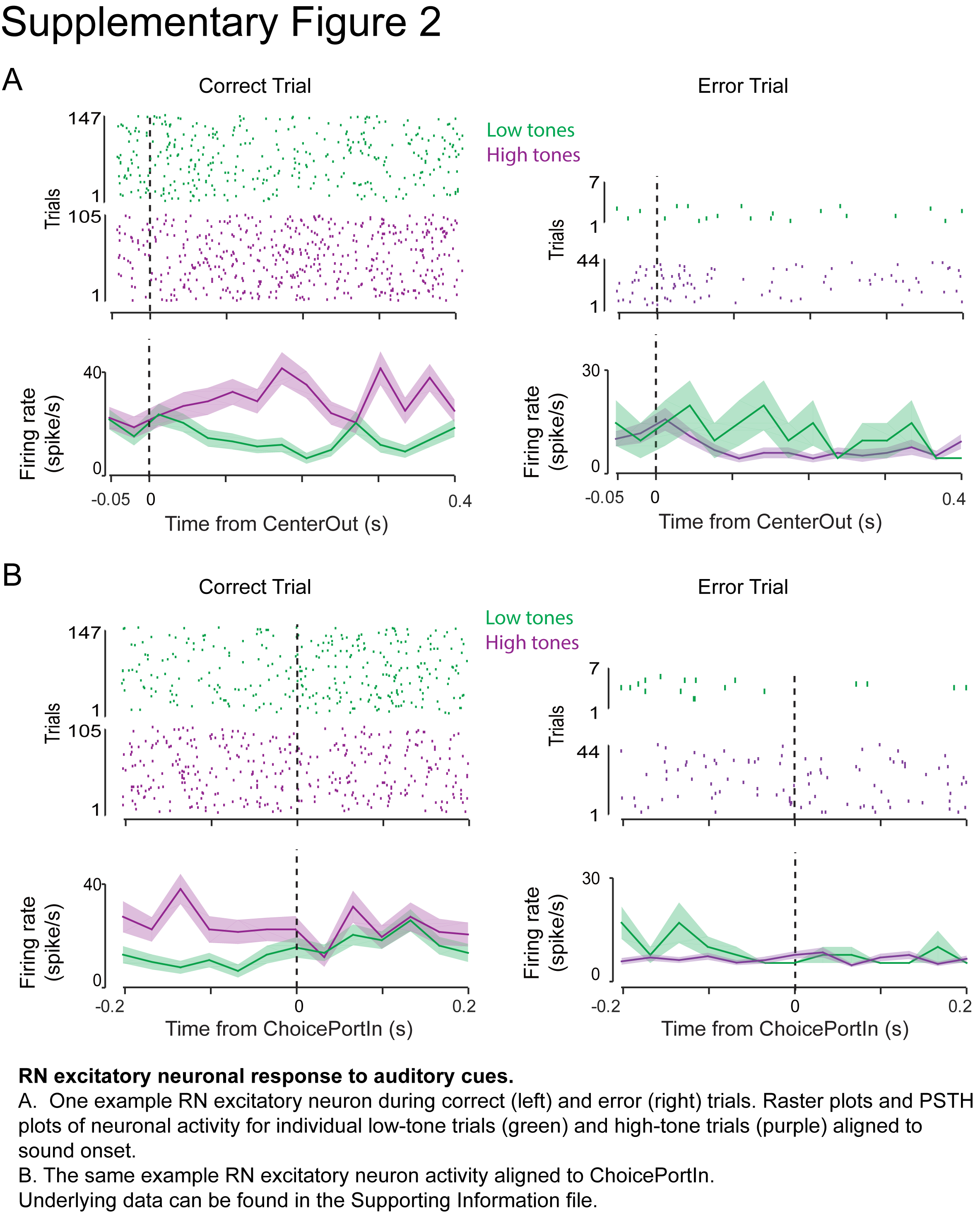

Supplement: S2 Fig — A. One example RN excitatory neuron during correct (left) and error (right) trials. Raster plots and PSTH plots of neuronal activity for individual low-tone trials (green) and high-tone trials (purple) aligned to sound onset. B. The same example RN excitatory neuron activity aligned to ChoicePortIn. Underlying data can be found in the S1 Data. (TIF) [file pbio.3003092.s002.tif]

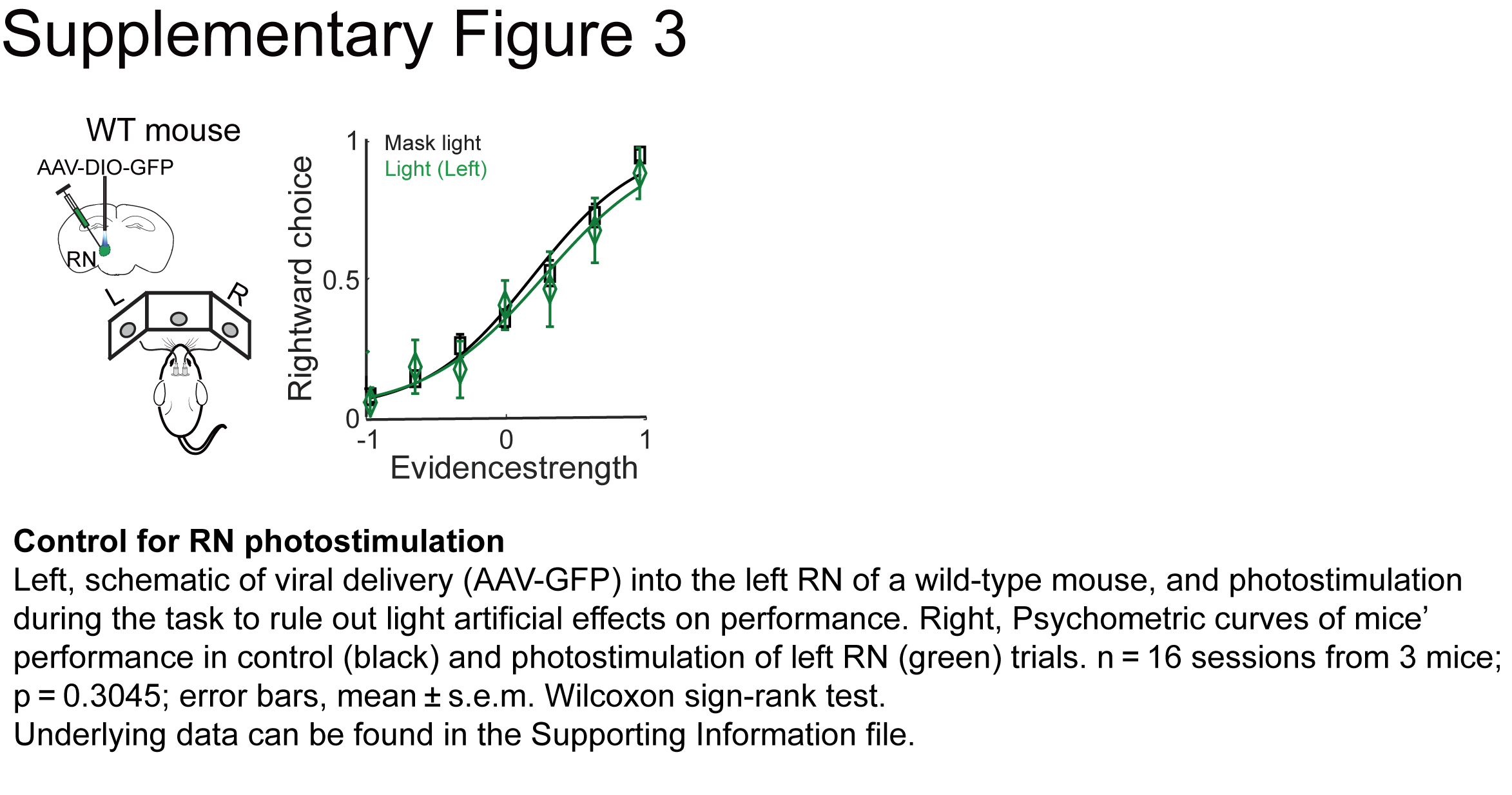

Supplement: S3 Fig — Left, schematic of viral delivery (AAV-GFP) into the left RN of a wild-type mouse, and photostimulation during the task to rule out light artificial effects on performance. Right, Psychometric curves of mice’ performance in control (black) and photostimulation of left RN (green) trials. N == 16 sessions from 3 mice; p == 0.3045; error bars, mean ±± s.e.m. Wilcoxon sign-rank test. Underlying data can be found in the S1 Data. (TIF) [file pbio.3003092.s003.tif]

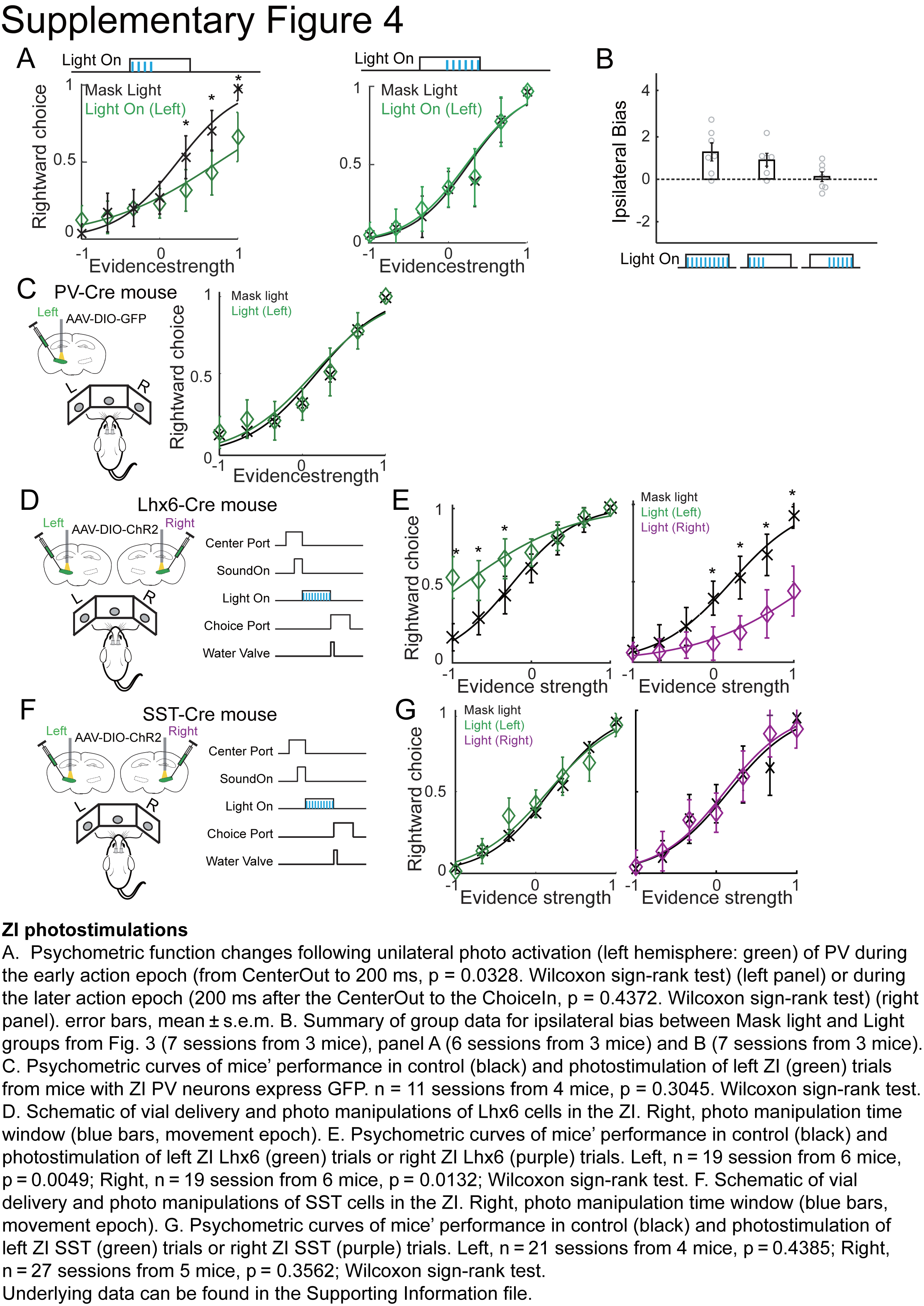

Supplement: S4 Fig — A. Psychometric function changes following unilateral photo activation (left hemisphere: green) of PV during the early action epoch (from CenterOut to 200 ms, p == 0.0328. Wilcoxon sign-rank test) (left panel) or during the later action epoch (200 ms after the CenterOut to the ChoiceIn, p == 0.4372. Wilcoxon sign-rank test) (right panel). Error bars, mean ±± s.e.m. B. Summary of group data for ipsilateral bias between Mask light and Light groups from Fig 3 (7 sessions from 3 mice), panel A (6 sessions from 3 mice) and B (7 sessions from 3 mice). C. Psychometric curves of mice’ performance in control (black) and photostimulation of left ZI (green) trials from mice with ZI PV neurons express GFP. N == 11 sessions from 4 mice, p == 0.3045. Wilcoxon sign-rank test. D. Schematic of vial delivery and photo manipulations of Lhx6 cells in the ZI. Right, photo manipulation time window (blue bars, movement epoch). E. Psychometric curves of mice’ performance in control (black) and photostimulation of left ZI Lhx6 (green) trials or right ZI Lhx6 (purple) trials. Left, n == 19 session from 6 mice, p == 0.0049; Right, n == 19 session from 6 mice, p == 0.0132; Wilcoxon sign-rank test. F. Schematic of vial delivery and photo manipulations of SST cells in the ZI. Right, photo manipulation time window (blue bars, movement epoch). G. Psychometric curves of mice’ performance in control (black) and photostimulation of left ZI SST (green) trials or right ZI SST (purple) trials. Left, n == 21 sessions from 4 mice, p == 0.4385; Right, n == 27 sessions from 5 mice, p == 0.3562; Wilcoxon sign-rank test. Underlying data can be found in the S1 Data. (TIF) [file pbio.3003092.s004.tif]

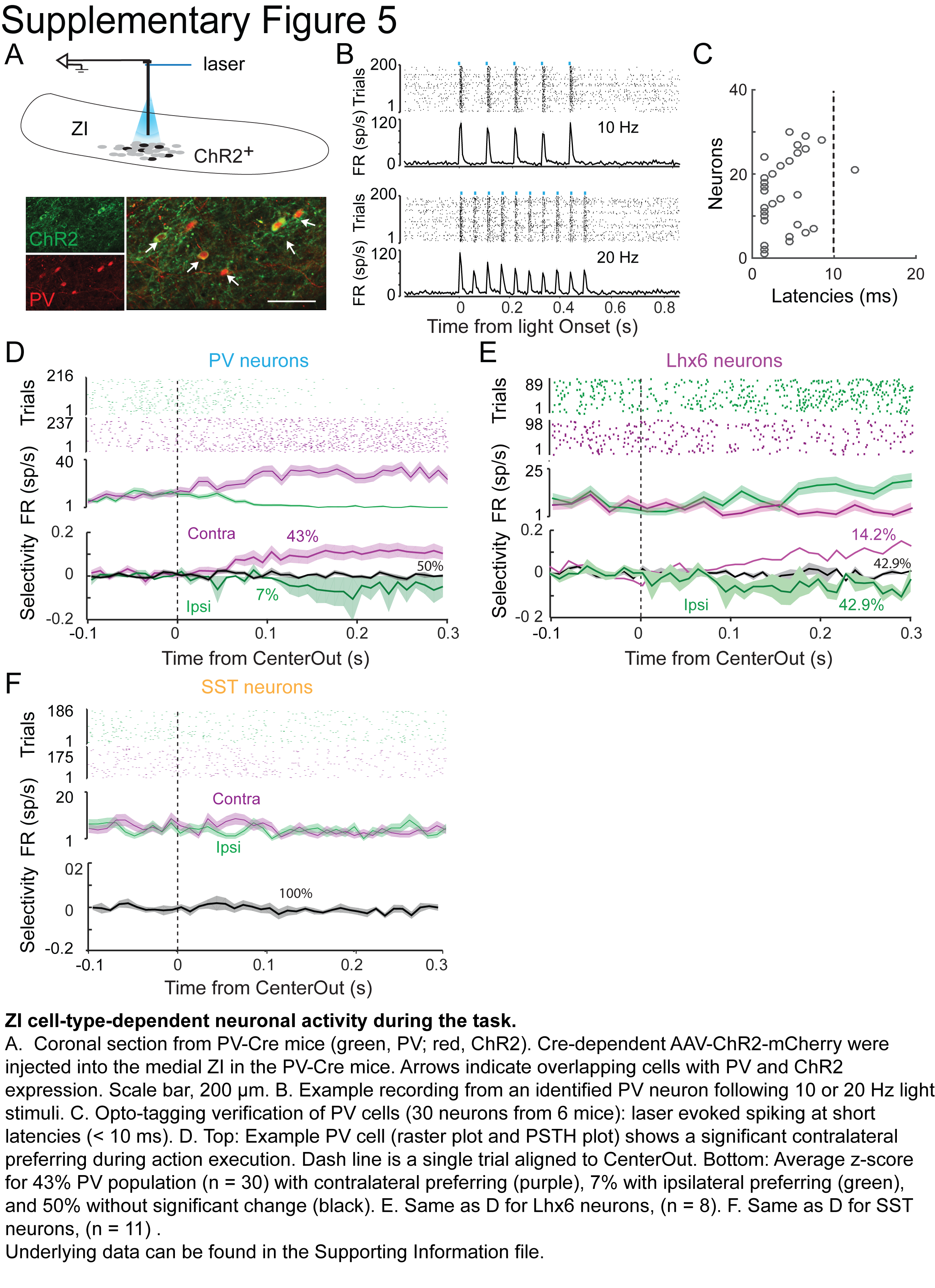

Supplement: S5 Fig — A. Coronal section from PV-Cre mice (green, PV; red, ChR2). Cre-dependent AAV-ChR2-mCherry were injected into the medial ZI in the PV-Cre mice. Arrows indicate overlapping cells with PV and ChR2 expression. Scale bar, 200 μm. B. Example recording from an identified PV neuron following 10 or 20 Hz light stimuli. C. Opto-tagging verification of PV cells (30 neurons from 6 mice): laser evoked spiking at short latencies (< < 10 ms). D. Top: Example PV cell (raster plot and PSTH plot) shows a significant contralateral preferring during action execution. Dash line is a single trial aligned to CenterOut. Bottom: Average z-score for 43% PV population (n == 30) with contralateral preferring (purple), 7% with ipsilateral preferring (green), and 50% without significant change (black). E. Top: Example Lhx6 cell (raster and PSTH plot) shows a significant ipsilateral preferring during action execution. Dash line is a single trial aligned to CenterOut. Bottom: Average z-score for 42.9% Lhx6 population (n == 8) with ipsilateral preferring (green), 7% with contralateral preferring (purple) and 50% without significant change (black). F. Top: Example SST cell (raster and PSTH plot) shows a non- significant preferring during action execution. Dash line is a single trial aligned to CenterOut. Bottom: Average z-score for 100% SST population (n == 11) without significant change (black). Underlying data can be found in the S1 Data. (TIF) [file pbio.3003092.s005.tif]

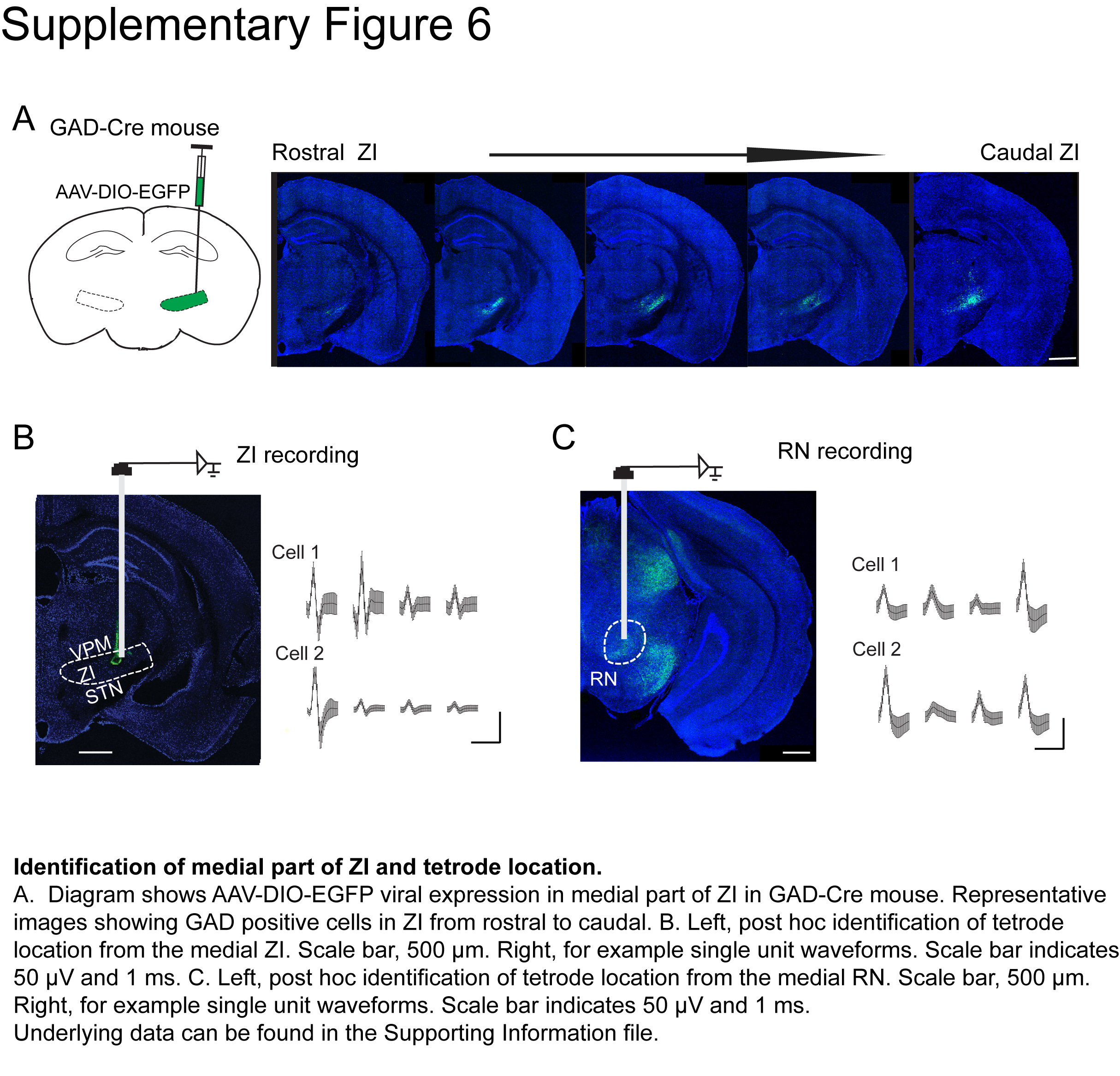

Supplement: S6 Fig — A. Diagram shows AAV-DIO-EGFP viral expression in medial part of ZI in GAD-Cre mouse. Representative images showing GAD positive cells in ZI from rostral to caudal. B. Left, post hoc identification of tetrode location from the medial ZI. Scale bar, 500 μm. Right, for example single unit waveforms. Scale bar indicates 50 μV and 1 ms. C. Left, post hoc identification of tetrode location from the medial RN. Scale bar, 500 μm. Right, for example single unit waveforms. Scale bar indicates 50 μV and 1 ms. Underlying data can be found in the S1 Data. (TIF) [file pbio.3003092.s006.tif]

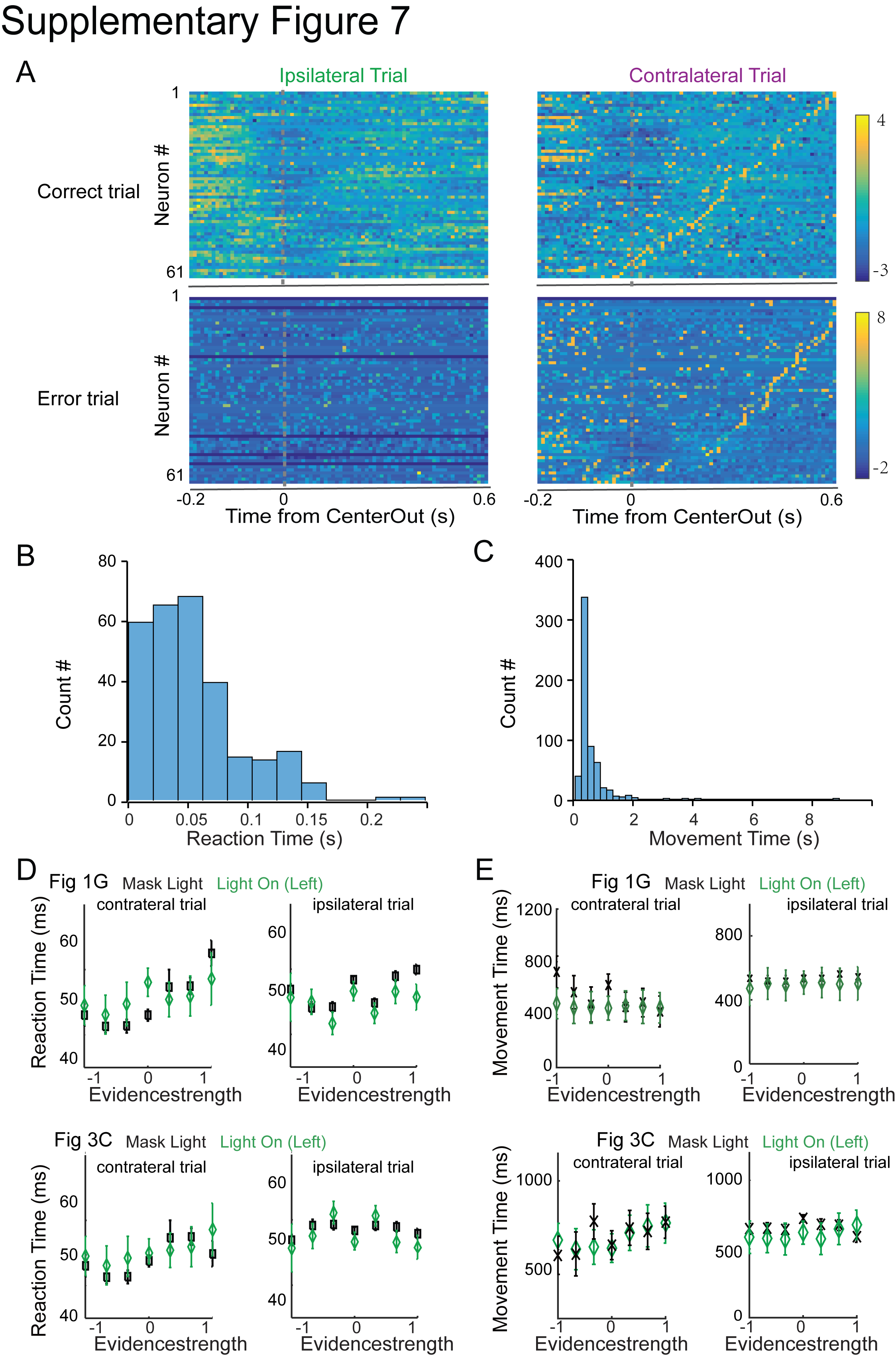

Supplement: S7 Fig — A. Heatmap showing a higher neural activity for contralateral preference during correct (upper) and error trials (lower). The color scale represents neural activity, with warm colors indicating higher activity and cool colors indicating lower activity. B. Histogram illustrating the distribution of reaction times across the entire session, with an average reaction time of 0.05 s. C. Histogram depicting the distribution of movement times from the entire session, with an average movement time of 0.8 s. D. Scatter plot showing reaction time from both contralateral trials and ipsilateral trials in Fig 1G (upper) and Fig 3C (lower) in photo-stimulation (green) and control conditions (black). E. Scatter plot showing movement time from both contralateral trials and ipsilateral trials in Fig 1G (upper) and Fig 3C (lower) in photo-stimulation (green) and control conditions (black). Underlying data can be found in the S1 Data. (TIF) [file pbio.3003092.s007.tif]

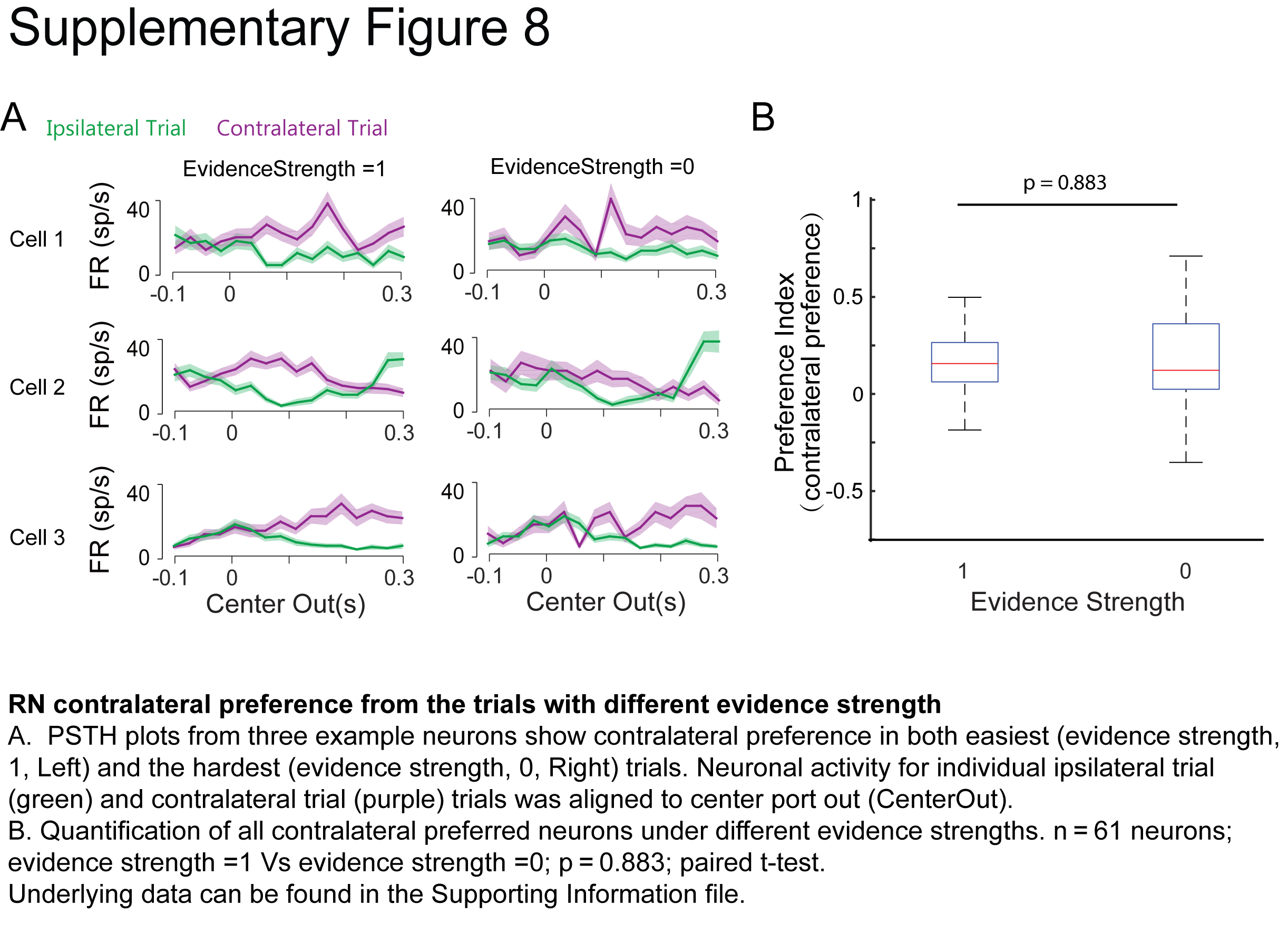

Supplement: S8 Fig — A. PSTH plots from three example neurons show contralateral preference in both easiest (evidence strength, 1, Left) and the hardest (evidence strength, 0, Right) trials. Neuronal activity for individual ipsilateral trial (green) and contralateral trial (purple) trials was aligned to center port out (CenterOut). B. Quantification of all contralateral preferred neurons under different evidence strengths. n == 61 neurons; evidence strength == 1 Vsversus evidence strength == 0; p == 0.883; paired t- test. Underlying data can be found in the S1 Data. (TIF) [file pbio.3003092.s008.tif]
