# Supplementary material for: The zona incerta negatively regulates the red nucleus during movement cued by sound signals
Source: PLoS Biol. 2025 Apr 7;23(4):e3003092. doi: 10.1371/journal.pbio.3003092 (PMC12002635; doi:10.1371/journal.pbio.3003092)
Supplement: Attachment — Submitted filename: Response to reviewers.pdf [file pbio.3003092.s010.pdf]

We thank the reviewers and editor for their careful reading of the manuscript and their constructive remarks. We have taken the comments on board to improve and clarify the manuscript. Please find below a detailed point-by-point response to all comments (reviewer's comments in grey, our reply in black).

Reviewer #1: The present study explored the role of the zona incerta and red nucelus in orchestrating the movements associated with auditory-guided decision making. The paper is easy to read and the results are clear, and the question is interesting and not well studied. However, the inclusion of additional details and control experiments would greatly strengthen the paper. Comments and suggestions are provided below.

1. Major:1. Optogenetic activation of ZI-originating PV+ axon terminals in the RN suppressed neuronal activity in the RN (Fig 3A). The authors interpret this observation as evidence that the monosynaptic PV+ connection between the ZI and RN inhibits RN neurons. While this is the most parsimonious explanation, it's also possible that optogenetic stimulation of the axon terminals led to antidromic activation of the PV+ cell bodies in the ZI. If these neurons project to other downstream targets that, in turn, also project to the RN, then it's possible that the effects observed are mediated by a di-synaptic, or even a multi-synaptic pathway, rather than a monosynaptic connection. Do PV+ cells in the ZI make connections with other downstream targets? If so, this issue could be resolved by repeating the experiment while pharmacologically silencing the ZI.

We agree with the reviewer that the observed suppression of RN activity in the original Fig 3A could be due to indirect effects of ZI-PV activation. To rule out the indirect connection possibility, we applied TTX in ZI to block antidromic activation of PV neurons while activating ZI-PV terminals in the RN, as suggested by the reviewer. We found that, majority of RN neurons (52%) are inhibited by PV-ChR2 terminal activation (tetrode recording results). We have updated this result to the new figure (**Fig 3A&B**) and text (Result section page 8 lines 16-22 to page 9 lines 1-6, Method section page 18 line 23 to page 19 lines 1-2).

2. Optogenetic activation (or suppression) of PV+ ZI neurons resulted in an ipsilateral (or contralateral) movement bias (Fig 3B-D). The authors interpreted these data as evidence that the monosynaptic PV+ connection between ZI and RN regulates movement during the task. However, in this experiment, PV+ cells were manipulated at the level of the cell body, not the axon terminal. Similar to my comment above, this leaves open the possibility the effects are mediated by other downstream targets of ZI PV+ neurons. If PV+ cells in the ZI do project to other brain regions, this issue could be resolved by repeating the experiment, but applying optogenetic manipulations to the ZI-originating PV+ axon terminals in the RN, rather than at the cell body (and controlling for antridromic effects as suggested above).

We agree with the reviewer on this concern. Using the same approach as above by 1) applying TTX in right-side ZI, and 2) photo stimulating ChR2+ ZI-PV terminals in the right-side RN, we repeated the behavioral test. As shown in new **Figure 3F**, our new results demonstrate the same behavioral effect – activating ZI-PV terminals in RN induces ipsilateral bias in choice. We have updated this result to the latest figure (**Fig. 3**) and text (result section page 9 lines 17-22 to page 10 lines 1-2, Method section page 18 line 23 to page 19 lines 1-2).

3. A major question I was left with while reading the manuscript was how specific these results are to the execution of goal-directed movement. For example, if you stimulate PV+ ZI cells or CaMKII+ RN cells outside of the context of the task, do you still generate laterally biased movements? Similarly, do RN neurons exhibit side preferences during spontaneous exploratory movements outside of the context of the task? It seems as though the authors either need to perform additional experiments to answer these questions or

include a more thorough presentation of the existing literature to address these questions in the discussion. (In its current form, the discussion section is far too short and feels somewhat superficial anyway).

We appreciate the reviewing for pointing out this caveat between our original data set and the conclusion. We performed an additional experiment to clarify the point of goal-directed movement. When we photo stimulating ZI-PV terminals (ChR2+) in the RN out of the task context, we did not observe significant directional movement effect (**Fig. 3G**), indicating that it is task specific. Interestingly, even within the task, if we photo activating ZI-PV neurons 200 ms after the onset of movement, there was no effect on performance (**Suppl. Fig. 4A&B**). The total movement duration from center port to choice port mainly ranges from 400 to 800 ms, with the average of 500 ms (**Suppl. Fig. 7C**). For both contralateral and ipsilateral trials, photo stimulation of ZI PV neurons or RN neurons did not change the movement durations (**Suppl. Fig. 7E**). Altogether our findings suggest that this ZI-RN activity is specific to goal-directed movement. We have updated the results in **Figure 3** and **Supplementary Figure 4 and Supplementary Figure 7**, and the text (page 10 lines 9-12).

We also included more discussions on RN roles in goal-directed movement based on current literature and our findings (page 13 lines 16-22, page 14 lines 15-22 to page 15 lines 1-2).

4. The paper would benefit from the inclusion of additional evidence to support some of the claims made. For example:

-The authors show that injection of AAV-DIO-GFP into the ZI of Lhx6- and SST- mice failed to label axon terminals in the RN (Fig 2E), but do not show images verifying that the injections were successful and that the viral expression was limited to ZI. The authors should add images showing the injection sites and labeled cell bodies in the ZI of these mice.

We agree with the reviewer and have added representative images showing the injection sites in the ZI of PV-Cre, Lhx6-Cre, and SST-Cre mice in **Figure 2E**.

-For the tetrode recordings, it would be helpful to see representative histological images confirming that the electrode tracks successfully hit their intended target. It would also be helpful to see representative voltage traces to get an idea of the recording quality/SNR. (Isolated, sorted spikes alone don't provide that information).

We have included representative images showing the tetrode tracks in the RN and ZI, and example waveforms of isolated single units (**Suppl Fig. 6B&C**).

5. The text that refers to Figure 4K reads "…Lhx6 and PV neurons in ZI may integrate auditory decision cues and then impact the RN activity to regulate the movement…" However, Figure 4K seems to depict a different scenario, in which auditory decision cues are integrated in the RN, not the ZI. The authors should clarify their working model.

We thank the reviewer for pointing out this confusion. We have revised the text accordingly to match the text and the figure model. (page 13 lines 2-4).

6. The authors suggest that ZI PV+ neurons "counteract the movements directed by auditory decisions." Another possibility is that the Lhx6-> PV-> RN disinhibitory circuit actually mediates these movements. Resolving this question would require recordings from Lhx6+ and PV+ neurons in the ZI during task performance. If they are truly counteracting movements directed by auditory decisions, we'd expect that

during such movements, Lhx6+ neurons would be relatively quiet, and PV+ neurons would be active. Alternatively, if the circuit is actually guiding the movements, then we'd expect the opposite (high Lhx6+ activity, low PV+ activity during the movement). The authors should consider performing these experiments. If such an experiment is not feasible due to the relatively low-yield or technical challenges involved, is there existing literature to draw from to make an educated guess about the activity of these neurons during behavior? This issue should be discussed more thoroughly, regardless.

We agree with the reviewer and have performed the suggested recording. Using ChR2-assisting tagging approach (Lima, S. et al. PLoS One, 2009), we identified PV, SST, Lhx6 neurons in the ZI through tetrode recording, and analyze their activity patterns during task performance (**Suppl. Fig. 5**). We found that:

1) 43% of identified PV neurons (13 out of 30) displayed contralateral movement preference, 7% (2 out of 30) displayed ipsilateral movement preference, and the rest (15 out of 30) with no preference.

2) 3 out of 7 identified Lhx6 neurons displayed ipsilateral movement preference, 1 out of 7 displayed contralateral preference, and the rest 3 with no preference.

3) All of the identified SST neurons (n=11) displayed no preference.

Combined with our optogenetic manipulation results (**Fig. 3**), we concluded that both Lhx6 and PV neurons in the ZI are activated during the task performance, and their activities together counteract the movements directed by auditory decisions.

We have updated these results to **Supplementary Figure 5** and text (result section page 11 lines 1-13, method section page 19 lines 14-23 to page 20 lines 1-5)

Minor:
1. The discussion and the methods section refer (in multiple places) to anterograde tracing from the auditory cortex and/or auditory striatum, but these experiments are not presented in the results section. Please correct this discrepancy.

We removed these sections.

2.The methods section refers to a "long-term delay task" in which nose poking triggered a light. This is the first and only mention of this task. Does it differ from the behavioral task presented in the manuscript? Is the light illumination referring to optogenetic stimulation or the house lights? Or an LED in the nose poke? Please clarify.

We removed this text to correct this mistake.

3.There's an unformatted reference Fogarty, 2007 #51 in the methods section.

We updated the reference.

4.The sound intensity is listed as 65 dB. dB relative to what? dB SPL? Please clarify.

We have updated the text to dB SPL.

5. I assume that "stimulus strength r" (methods) and "evidence strength" (figures) are synonymous. If so, please pick one term to use consistently. If not, please clarify.

We have revised the method text to “evidence strength”.

6. Please describe the quantitative criteria used to identify single units.

We have included the criteria used to determine single units in the method section (page 18 lines 12-13): Clusters with isolation distance >20 and L-Ratio <0.1 were included.

7. More details are needed regarding the optogenetic stimulation parameters. (Length of stimulation, pulse width, pulse rate, etc).

We have included more details of optogenetic stimulation in the method section: for ChR2-mediated activation (blue light 480 nm; 5 ms light pulse at 20 Hz), ArchT-mediated silencing (Orange light 530 nm; continuous). The stimulation durations vary in different experiments, we have included the specific durations in corresponding figure panels and legends (**Fig 1F**, **Fig 3B**, and **Fig 4B&I&J**).

Reviewer #2: Chen et. al., analyzed a neuronal circuit involving projections of zona incerta to the red nucleus which modulates movement directed by an auditory signal. The authors describe that Parvoalbumin positive interneurons of the zona incerta inhibit red nucleus, modulating movement decision-making directed by auditory signal. The results are interesting and show original knowledge about subcortical structures' role in motor control that has been poorly studied and abandoned for several decades. There are some aspects of the manuscripts and the way the results are presented that deserve to be attended.

General concerns:

How specific is the zona incerta-red nucleus neuronal circuit for auditory signal-cued movements? Is not justified in the introduction if the circuit is selective for auditory goal-directed tasks or could be relevant for motor control in general.

In fact, the 2nd paragraph of the results section "Red Nucleus receives projection of parvalbumin-expressing neurons in the Zona Incerta" could be part of the introduction to better understanding the state of the art and the objectives of the study.

We assume that the "introduction" refers to the discussion section, and completely agree with the reviewer that whether the circuit is specific for goal-directed movement is an important aspect. In the revision, we included new sets of analyses to test whether ZI PV-RN pathway is for general motor control. First, we showed that photo stimulation of ZI PV neurons during the initiation period of the movement (0-200 ms from center port withdrawal) but not the later phase (200 ms to the end) induced behavioral bias (**Suppl. Fig. 4A&B**). RN maintains contralateral preference during both the correct and error trials (**Suppl fig 7A**). The total movement duration from the center port to the choice port mainly ranges from 400 to 800 ms, with an average of 500 ms (**Suppl. Fig. 7C**). Second, for both contralateral and ipsilateral trials, photo-stimulation of ZI PV neurons or RN neurons did not change the movement durations (**Suppl. Fig. 7E**). Third, we found that photo-stimulation of ZI PV neurons outside the task did not induce any directional preference (**Fig. 3G**). Together, our results indicate that the ZI PV-RN pathway is involved in goal-directed movement coordination, rather than general motor control. We have included the new analysis in the result section (page 9 lines 13-14, page 10 lines 9-12), as well as the discussion section (page 13 lines 16-22, page 14 lines 15-22 to page 15 lines 1-2).

Results:

1) Are the data collected from correct trials? What happened in incorrect trials? i.e. when the animals move to the left or right without any relation to the movement choice

We assume that the "data" here refers to the tetrode recording and analyses of RN neuronal activity during task performance. In **Figure. 1C-E**, we only included correct trials. In the new **Supplementary Figure 7A**, we added the comparison between correct and error trials. This is a two-alternative-force choice task, in which mice will move to one of the two side ports to complete the trials (water reward for correct trials, timeout for error trials). We found the RN neurons showed the same activity patterns in both correct and error trials when they moved to the same sides, indicating that the RN neuronal activity is correlated with the side of the movement (movement decision) rather than auditory cue identity (sensory representation). We have included the new analyses in the revision (**Suppl. Fig. 7A**, result section page 5 lines 18-21).

2) Despite the optogenetic activation bias the choice, is necessary to test if RN activity modulates the choice or only triggers the movement.

We agree with the reviewer that this point is important. As we described in response to the general concern above, the three lines of evidence (1, ZI PV photo activation is only effective in the early but not late phase of movement; 2, no effect in movement time; 3, no effect outside the task) suggest that this ZI-RN pathway modulates the choice of direction rather than simply triggers the direction of movement. We have included these results in the revised **Supplementary Figure 4A&B**, **Supplementary Figure 7E**, and **Figure 3G**, respectively. Result section page 9 lines 13-14, page 10 lines 9-12, and the discussion section page 13 lines 16-22, page 14 lines 15-22 to page 15 lines 1-2.

3) The readers would appreciate some histology nice confirmation images from recording and injection sites.

We thank the reviewer for pointing it out. We have included representative brain slice images to demonstrate the viral injection sites and tetrode recording sites in **Figure 2E** and **Supplementary Figure 6B&C**.

4) Last paragraph of the section "Red Nucleus receives a projection of parvalbumin-expressing neurons in the Zona Incerta" : Did we find? It does not sense, do you mean "were expressed?"

We are sorry for the typos that cause the confusion. We have revised the text to "We found that there were GFP+ terminals in the ipsilateral RN (indicating projections from ZI) only from the PV-Cre mice. In the Lhx6- and SST-Cre mice, we did not detect GFP+ signal in the RN (**Fig. 2E**). Together our results show that ZI PV neurons, but not Lhx6 and SST neurons, project to the RN."

5) First paragraph of the section "Activation of parvalbumin-expressing neurons in the Zona Incerta inhibited RN neurons and biased the movement choices in the auditory task" : Among the 65 identified units, 29 of them dramatically decreased firing…..what happened with the other neurons?

We have revised the experiment according to other reviewers' comments: to rule out the possible antidromic effect of ChR2 activation, we infused TTX into ZI during the recording. The updated result is shown in **Figure 3A&B**, and the description is at page 8 lines 16-22 and page 9 lines 1-4: "As illustrated in Figure 3A, we expressed ChR2 in PV neurons of the left ZI by injecting an AAV carrying DIO-ChR2, a Cre-dependent expression cassette of ChR2, into left ZI of PV-Cre mouse. A cannula was implanted right above the left ZI. A tetrode bundle integrated with an optical fiber, termed optrode, was implanted in the left RN. 3 weeks after the surgery, neuronal activity of the left RN upon optical stimulation was recorded using the tetrode method as previously described. To prevent the interference of antidromic activation of ZI PV soma, we infused TTX (1 μM) through the cannula in ZI during the recording. We compared the firing rates between baseline (50 ms before the light onset) and light period (0-500 ms) for each recorded neuron, using Wilcoxon rank-sum test. Among the 44 identified units, 23 of them dramatically decreased firing rates in response to the light stimulation, 3 of them increased the firing rates and the rest 18 units showed no detectable changes in firing rates (**Fig. 3A&B**). Overall, it indicates that the ZI PV neuron activation suppressed the neuronal activity in RN."

Methods:
Some details are necessary: how many trials did the mice have in each session? What was the recording frequency used? How many mice were used for each experiment?

We have included the information in the result and method section: 1) mice completed 600-1000 trials for each session; 2) the tetrode recording used 30 kHz sample frequency, optogenetic stimulation in ChR2 experiments are at 20 Hz; 3) animal numbers used in each experiment are included in each figure legend.

Reviewer #3: This paper sets out to better understand neural correlates in Red Nucleus (RN) and how upstream signals from Zona Incerta (ZI) impact firing in RN using a variety of techniques (recording, opto, and tracing) in mice performing a 2-choice auditory motor task. The author replicates previous work in rats showing activity is stronger in RN during contralateral movement and further demonstrates that stimulating RN promotes contralateral movement. Using a variety of techniques they then go on to claim that ZI to RN works as a brake by showing that connections exist, ZI inhibits RN and that ZI perturbation alters contra/ ipsi preference. The use of multiple techniques is impressive but I have many concerns about the paper. I think this could be a very good paper if they address all the issues below.

1. -I'm concerned that the authors did not counterbalance auditory cues across mice. Motor directional responding might reflect tone frequency. -Instead of counterbalancing to rule out auditory selectivity the authors do an analysis to determine if firing during presentation of the auditory cue is selective. That does not rule out that later firing is not related to the frequency of the stimulus. Also, in supplemental figure 2 there are no stats and preference is not adequately described. There is no way to determine if purple is different than green, which is the critical question. If I do a chi-square 15 is more than 4. The entire preference distribution also looks shifted to me but there is no stats. There should be stats for all distributions in the paper. Since 15 appears more than 4 and the distributions is shifted it is difficulty to use this analysis to rule out frequency selectivity. It is also not clear what the analysis epoch was or how many mice were used in this analysis.

We thank the reviewer for pointing out this confusion. We revised the text and figure/figure legend accordingly to address this concern. We also added animal number information for all related analyses.

Indeed, because the tone frequency is associated with the reward side that directing the movement direction, it is possible that the contralateral movement preference we showed in **Figure 1** is due to the delayed sound frequency preference. To rule out this possibility, we compared the neuronal activities and their direction preferences between correct trials and error trials. In contralateral correct trials and contralateral error trials, the mice heard high-frequency tones and low-frequency tones, respectively, and moved toward the contralateral (rightward) side port. If the preference is to the sound frequency, we should see different firing patterns; if the preference is to the movement direction, we should see the same firing pattern. As shown in **Supplementary Figure 1D** and **Supplementary Figure 7A**, we found the latter result --- in both correct and error trials when mice moved to the same direction, the RN neurons fire in the same pattern.

2. -For Supplemental figure 2 are these correct trials only? Also, why does it look like there is higher firing for low tones in A. These look normalized but not sorted like the figures in the main text.

We thank the reviewer for pointing out this confusion. In the original **Supplementary Figure 2** the neuronal activities are aligned to the sound onset, in **Figure 1** they are aligned to the movement onset (center out). Because for most of the neurons there is no significant response to sound, we did not sort them as we did for **Figure 1** (sorted based on the latency of the response after movement onset). Because the neuronal activity is normalized to low (ipsilateral) and high (contralateral) trials separately and there are many neurons having contralateral preference, the overall color tone seems to be higher in low trials than high trials within the presented time window. To avoid this confusion, in the revised **Supplementary Figure 2A**, we included one example plot of the same cell (**Fig. 1C**) activity aligned to SoundOn. We also included the plots showing both correct trials and error trials. We found that the neuronal activities are correlated with the movement directions (left or right) rather than the auditory stimuli (low or high tones).

3. -Error trials should be analyzed to determine if activity is tracking auditory stimuli or movement.

We agree with the reviewer and, as described above for #1 & #2, we included analyses for error trials in **Supplementary Figures 1&2**.

4. -Did firing track the different stimuli? That is, were neural directional signals stronger for stimuli at the extremes?

We thank the reviewer for this interesting question. We performed additional analysis to compare the direction preferences of neuronal activity between the trials with the strongest evidence strength (1 or -1) and the weakest evidence strength (0). As shown in **Supplementary Figure 8** for both example cells and population plots, there is no clear difference between the trials with different difficulties (evidence strengths).

5. -The task is very fast. The poke and tone presentation are less than 1 s. The reaction times and time to reward is not described. I assume reward is delivered immediately according to the task figure. With all these events happening so quickly in time the neural response might bleed into each other. Activity should be aligned to multiple events (eg, sound on, center out, choice, reward) so the readers can better interpret the data.

We agree with the reviewer. In the revised **Supplementary Figure 2** we added plots to show example alignments to SoundOn, and ChoiceIn (when the reward was immediately delivered). We also added the plot for reaction time in **Supplementary Figure 7B**, showing that most reaction times are around 50 ms. Together we found no strong indications of tone responses or reward responses from these alignments.

6. -To me it looks like half of the 61 neurons in Fig. 1E will be more related to port entry and reward delivery that the behavioral response, but it will be hard to tell because they happen so close in time and different alignments are not presented.

We thank the reviewer for pointing out this concern. In the revised **Supplementary Figure 7B&C**, we included quantifications for reaction time, and movement time (from center out to choice port poking). The minimal movement time is about 364 ms, which is beyond the time window we showed in **Figure 1E** (300 ms) demonstrating the movement-related activity. Together with the alignments we included in the revised **Supplementary Figure 2**, we hope it clears up the confusion.

7. -For figure 1C it would be nice to see the purple and green lines converge and to know when the movement ended and reward was delivered.

Because mice had different movement durations for individual trials, to answer this question we aligned the neuronal activity shown in Figure 1C to the time points of choice port poking (ChoicePortIn). As shown in **Supplementary Figure 1C and Supplementary Figure 2B**, the activity difference between ipsilateral and contralateral trials merged at the end of the movement (Choice Port In).

8-For figure 1 E it looks like there are increases in activity immediately before 0 for ipsilateral trials that are not present during contralateral, but we only see out to -0.05. The x-axis should be extended to at least -0.01 as in C, but ideally for the time the mouse imitated the trial.

We have expanded the time window to -0.1 s as shown in revised **Figure 1E** and **Supplementary Figure 7A**. As illustrated in **Figure 1B**, when trial starts at CenterIn, 0.05s later sound cues are on, the range of

reaction time (SoundOn to CenterOut) is around 0.05-0.15s. Because different trials have different lengths from the CenterIn to CenterOut, to avoid confusions about signals from other events, we did not show too long extension in x axis.

9. -There are no stats for figure 1D.

-It is not clear how many trials were performed.

-The regression model for behavior is not clear.

We thank the reviewer for pointing out this missing information. We added statistics and numbers to all relevant figure legends.

We also included the logistic regression model in the method section. $\log(p/(1 - p)) = \beta 0 + r^*\beta 1$, where p is the fraction of choices towards the port associated with high frequencies. Parameters β0 and β1 measure the bias and slope of the psychometric curve. Page 17 lines 16-19.

10. -It is not clear what mask light is.

We thank the reviewer for pointing out this missing information. To control for the potential influence of visual stimulation effects, during all trials, we included a masking light stimulation using the same presentation time window and wavelength as photo stimulation trials delivered through a bulb placed above the center port, as previously employed (Znamenskiy and Zador 2013, Chen, Malgady et al. 2022). We have included it in the method section (page 19 lines 9-12).

11.-For each behavioral analysis I would like to see the percent correct, reaction time and movement time for contra and ipsi movements with and without opto to have a better feel for the raw data. It would also be good to know how many trials were not completed due to opto.

In the new **Supplementary Figure 7D&E**, we separated the quantification of reaction time and movement time for contralateral and ipsilateral trials. (For both Figure 1 RN and Figure 3 ZI) There is no significant difference in these parameters between control and photo stimulation conditions. In addition, the mice have completed all trials during photo stimulation.

12.-Fig. 1 F is not informative because there is no time scale bar.

We included a timeline of a single trial in the revised **Figure 1A**. The photo stimulation indicated in **Figure 1F** is from the beginning of center port withdrawal to the side port poking in, which varies across trials.

13.-Did stimulation for experiments shown if Fig 1G make them faster? Was it that they just got better at contralateral movements or were low tones that signaled ipsi movement also impacted?

We included the suggested analyses in the revised **Supplementary Figure 7D&E** (same as in #11). We did not observe detectable effect of photo stimulation on movement speed in either contralateral or ipsilateral directions.

14.-What are the counts of purple and green in fig. 1D? What are the stats?

We have included the counts in the revised figure legend: total 198 units, purple 61 units, green 13 units, Chi2stat = 38.2904; P = 6.096e-10).

15. -I don't understand why opto light is on during the time after the auditory cue. This would be after the time the mouse has made its decision. I guess that argument is that these brain areas are not important for the decision to move but the act and coordination of moving? If so, does this make the fact that this is an auditory task irrelevant and that authors are not study neural processes related to auditory decision making, but just movement in general?

We agree with the reviewer that the photo stimulation timing is after the auditory decision, but we wanted to test whether RN is involved in controlling this goal-directed movement. In the revised manuscript, we included additional experiment and analysis to test this goal-directed movement vs movement in general question.

First, when we photo stimulating ZI-PV terminals (ChR2+) in the RN out of the task context, we did not observe significant directional movement effect (**Fig. 3F**), indicating that it is task specific. Second, we found that even within the task, if we photo activating ZI-PV neurons 200 ms after the onset of movement, there was no effect on performance (**Suppl. Fig. 4A&B**). The total movement duration from center port to choice port mainly ranges from 400 to 800 ms, with the average of 500 ms (**Suppl. Fig. 7C**). Third, for both contralateral and ipsilateral trials, photo stimulation of ZI PV neurons or RN neurons did not change the movement durations (**Suppl. Fig. 7E**). Altogether our findings suggest that this ZI-RN activity is specific to goal-directed movement. We have updated the results in **Figure 3F**, **Supplementary Figure 4A&B**, and **Supplementary Figure 7C&E**. Result section page 9 lines 13-14, lines 17-22).

We also included more discussions on RN roles in goal-directed movement based on current literature and our findings (page 13 lines 16-22, page 14 lines 15-22).

16. -For each experiment it is not clear how many neurons were recording from in each mouse.

We added neuron numbers and mouse numbers to related individual experiments (page 5 lines 15-16).

17.-For many of the opto experiments there are multiple sessions that are conducted, but it is not clear how many come from each mouse. For example, there 16 sessions from 3 mice in figure 1G. How were these distributed across the 3 mice? This should be clear throughout.

We included more detailed number descriptions in the corresponding figure legends**: Figure 1G** and **Figure 3C&D**.

18.-It is not clear to me that these behaviors are "goal" directed behaviors and not just stimulus response driven behaviors (ie, habit).

This behavior is goal-directed because if we omitted the water reward, the mice quickly stopped performing the task within tens of the trials. We have included this information in the text for clarification (page 5 lines 8-9).

19.-There is some emphasis on the "auditory", but the opto perturbations occur after the auditory cue and after the decision to move has been made so it is not clear if this really has anything to do with the auditory cue driving firing in RN because the motor command has already been sent.

We thank the reviewer for pointing out this confusion. As we discussed in response to #15, we consider this ZI PV to RN pathway is involved in the goal-directed movement. We agree with the reviewer that it may not be auditory specific or sensory specific. We included this discussion on page 14 lines 15-22.

20.-There is no verification of virus and fiber locations for any of the experiments.

We have included representative validation images for viral injection (**Fig. 2E** and **Suppl. Fig. 6B&C**).

21.-The pictures are pretty in Figure 2, but there is no quantification. Also, it is not clear how many mice were used in this experiment.

We agree with the reviewer that **Figure 2** provides qualitative but not quantitative information. In the experiment shown in **Figure 2A&B**, we searched for brain regions projecting to RN; in the experiment shown in **Figure 2C-E**, we searched for ZI neurons projecting to RN. This information guided the subsequent investigation of ZI PV neurons in the behavior. Quantification of the tracing results would require precise measurement of starter cells and projection counting, which is out of the scope of the current study. We included mouse numbers in the figure legend to demonstrate reproductivity.

22.-Figure 3. There are no stats for figure 3A. It looks like half the neurons are excited (bottom half) and half are inhibited. How many animals was this done in? It also looks like when cells are inhibited, once the light turns off, they are excited. This could impact behavior. The fact that so many neurons are excited suggests that the statement that ZI inhibits RN as a brake is not well supported.

We thank the reviewer for pointing out this confusion. By incorporating the other comments regarding potential antidromic interference, we revised the experiment in **Figure 3A&B** to infuse TTX in ZI and included the following descriptions to clarify the analyses: "We compared the firing rates between baseline (50 ms before the light onset) and light period (0-500 ms) for each recorded neuron, using *Wilcoxon* rank-sum test. Among the 44 identified units, 23 of them dramatically decreased firing rates in response to the light stimulation, 3 of them increased the firing rates and the rest 18 units showed no detectable changes in firing rates (**Fig. 3A&B**)." Page 8 lines 16-22 and page 9 lines 1-6.

Indeed, some neurons showed offset responses to the photo stimulation. However, we do not think it affects the behavioral results in our analyses because of the following observations: 1) Activation of RN excitatory neurons induced contralateral bias (**Fig. 1G**), and activation of ZI PV neurons induced ipsilateral bias (**Fig. 3C**). These two sets of data match well with the notion that there is a substantial population of RN excitatory neurons decreased firing rates in response to ZI PV activation (**Fig. 3A&B**). 2) In another newly included analyses (**Suppl. Fig. 4A&B**), we showed that photo activation of ZI PV neurons at the beginning of movement (0-200 ms from center port withdrawal) but not the later phase (200 ms to the end) induced the same behavioral bias as we showed before (activating ZI PV neurons during the whole movement period). If the RN offset response impacts behavior, we would expect to see different results. We included a discussion in the revised text (page 14 lines 15-22).

23.-It is stated that "Among the 65 identified units, 29 of them dramatically decreased firing rates in response to the 500ms light stimulation (Fig. 3A)". What is the statistic here? How many were excited? Were the frequencies different?

Please see the response above for #22. We have revised the text and figure legend to include this information.

24.-Figure 3. Are movement times impacted? Are mice completing trials during inhibition?

Please see the response for #11 (**Suppl. Fig. 7E**), we separated the quantification of reaction time and movement time for contralateral and ipsilateral trials. For both Figure 1 RN and Figure 3 ZI photo-stimulation, there is no significant difference in these parameters between control and photo stimulation conditions. In addition, the mice have completed all trials during photo stimulation.

25.-It is nice that stimulation in ZI impacts RN but stimulating or silencing ZI in Figure 3B-D does not prove that behavior is altered through this circuit as suggested by the statement "We next explored whether such inhibition by ZI PV neuronal activation to the RN affects the movements to reward ports in the task." As the authors described in the text, ZI has been implicated in many functions and has many connections.

We agree with the reviewer that our original set of manipulation did not directly tackle the ZI-RN connection. To address this issue, we conducted a new experiment in which we expressed ChR2 specifically in the ZI PV neurons and implanted optic fiber into the RN for photo stimulation. To rule out the possible influence of antidromic activation, we also implanted a cannula above the ZI for TTX infusion. We found that photo stimulation of ZI PV terminals in the RN induced ipsilateral bias when mice were performing the task, which is similar to what we observed in ZI PV cell body stimulation. The detailed description of this experiment and analyses are included in the revised text (page 9 lines 17-22, page 10 lines 1-2) and **Figure 3F**.

26.-There is no scale bar in figure 4 C or G. It also looks like neurons are sorted by inhibitory firing in C and excitatory firing in G, which does not make for a fair comparison.

For both panels, neurons are sorted in the same way: from the strongest inhibition effect to the strongest excitation effect in response to photo stimulation.

27.-It is unclear what statistic is being used to determine if cells are ChR2+ or - in figure 4.

In the revised method section, we included the criteria for determining ChR2+ neurons based on previous publications (Lima, Hromadka et al. 2009, Pi, Hangya et al. 2013). To identify neurons with ChR2-mediated responses, we performed the following three analyses: 1) distribution of spiking timing; 2) Latency; 3) spike waveform correlation. A unit (neuron) was determined as ChR2-expressing if: 1) we can detect a reliable increase of spike precisely time-locked to the onset of light pulses at 20 Hz, 2) the light response has a latency less than 10 ms, and 3) the spike waveforms have correlation of more than 0.95. Page 19 lines 14-23 to page 20 lines 1-5.

28.-In Figure 4 if 2 of 72 are excited and 43 of 72 are inhibited or unchanged by light pulses, what are the other 27 doing? Also, it is stated that 43 out of the 72 units showed strong inhibitory effects but, as defined in the text, this includes unchanged neurons? Why not just tell us those that were significantly inhibited? Also, there is way of knowing if these are really PV expressing.

We revised the corresponding text and figure panels to clarify our findings. For the results shown in **Figure 4A-D,** among the 72 neurons recorded in this experiment, 2 are ChR2+ and 70 are ChR2-, based on our criteria (see detail above in response to #27). Among the 70 ChR2- neurons, 54 of them were strongly inhibited by photo stimulation. Due to the technical limitation, we do not know for sure the cell type of ChR2- neurons. We moved the speculation to the end of this section as a brief discussion.

29.-The authors say that only 1/88 are ChR2- but looking at the heatmap there seems to be more and there are many that showed no effect, which were included in the 43 for figure 4D? That is not a fair comparison. Also, since you are looking for inhibitory effects you should sort your heat map on inhibition not excitation so the two populations can be adequately compared.

Similar to the above, we revised the text and figure panel to clarify the quantification. **Figure 4A-D** and **Figure 4E-H** are two separated experiments: the first having Lhx6 ZI neurons expressing ChR2 and the second having PV ZI neurons expressing ChR2. Therefore, the numbers in panel **D & H** are not overlapped --- they are two separate recordings.

We hope the revised text clarifies the confusion: "Among the 88 recorded neurons, 8 of them were ChR2+ and 80 were ChR2-. In contrast to the Lhx6 activation experiment, when we activated ChR2 expressing ZI PV neurons, we found no obvious effect on ChR2 negative neurons in the ZI (**Fig. 4E-H**). Only one neuron showed a potential inhibitory response (**Fig. 4H lower panel**)."

30.-In Figure 4, if you are comparing counts of neurons and trying to claim differences between frequencies of neurons, there needs to be a statistic (e.g., chi-square).

We have included the statistics in **Figure 4D&H** legend. 2/72 with activation versus 43/72 with silencing; Chi2stat = 54.3354; P = 1.6909e-13; 8/88 with activation versus 1/88 with silencing; Chi2stat = 5.7379; P = 0.0166).

31.-In the abstract it mentions that ZI to RN demonstrate that ZI to RN function as a braking system but are they stopping or are they going in the other direction. I also feel like that is too strong of a statement. Yes, ZI has an impact on behavior and so does RN, but it was not shown that disconnection between the two impact behavior and in Figure 3A there looks to be lots of excitations too.

We agree with the reviewer that since our behavioral task is a two-alternative forced choice task, it is not clear whether ZI-RN acts as stopping or oppositely directing. We revised the sentence in the abstract as "… demonstrate an inhibitory circuit connecting the ZI to RN **contributing** in the auditory-cued goal-directed movement".

As we respond above for #22, among the 44 identified units, 23 of them dramatically decreased firing rates in response to the light stimulation, 3 of them increased the firing rates and the rest 18 units showed no detectable changes in firing rates (**Fig. 3A&B**). Overall, it indicates that the ZI PV neuron activation suppressed the neuronal activity in RN. (page 9 lines 3-6)

32. For the PV-Cre cohort, were there GFP terminals in contralateral RN also?

We did not observe contralateral projection of ZI-PV neurons from our post hoc validation.

Reference:

Chen, A. P. F., J. M. Malgady, L. Chen, K. W. Shi, E. Cheng, J. L. Plotkin, S. Ge and Q. Xiong (2022). "Nigrostriatal dopamine pathway regulates auditory discrimination behavior." Nat Commun **13**(1): 5942.
Lima, S. Q., T. Hromadka, P. Znamenskiy and A. M. Zador (2009). "PINP: a new method of tagging neuronal populations for identification during in vivo electrophysiological recording." PLoS One **4**(7): e6099.

Pi, H. J., B. Hangya, D. Kvitsiani, J. I. Sanders, Z. J. Huang and A. Kepecs (2013). "Cortical interneurons that specialize in disinhibitory control." Nature **503**(7477): 521-524.
Znamenskiy, P. and A. M. Zador (2013). "Corticostriatal neurons in auditory cortex drive decisions during auditory discrimination." Nature **497**(7450): 482-485.
