# Supplementary material for: The zona incerta negatively regulates the red nucleus during movement cued by sound signals
Source: PLoS Biol. 2025 Apr 7;23(4):e3003092. doi: 10.1371/journal.pbio.3003092 (PMC12002635; doi:10.1371/journal.pbio.3003092)
Supplement: Attachment — Submitted filename: Response_to_reviewers_auresp_2.pdf [file pbio.3003092.s012.pdf]

We thank the reviewers and editors for their constructive remarks. We have taken the comments on board to improve and clarify the manuscript. Please find below a detailed point-by-point response to the remaining comments.

Reviewer #1: The article has improved significantly; the results are now easy to read, and the analysis and experiments are presented logically. However, the authors' path to arrive at the main question remains somewhat elusive. Although the question posed is clear, the hypothesis lacks effective articulation. A well-defined hypothesis should outline a clear cause-and-effect relationship, but unfortunately, this structure is not apparent, leaving the reader grappling with ambiguity.

In this way, in the introduction section, the role of ZI appears abruptly and feels disconnected from the overall narrative. Without adequate context and integration into the hypothesis, the significance of ZI becomes questionable.

In summary, while the article has made notable strides in clarity, improving the articulation of the hypothesis and providing a better context for ZI would greatly enhance the overall coherence and impact of the paper.

We thank the reviewer for this thoughtful suggestion and agree with this comment. In the revision, we have revised the abstract and the introduction sections (both yellow-color highlighted) to clarify the inclusion of the ZI and the hypothesis of ZI-RN circuit in movement modulation.
